# An Asynchronous Multi-Sensor Micro Control Unit for Wireless Body Sensor Networks (WBSNs)

**DOI:** 10.3390/s110707022

**Published:** 2011-07-06

**Authors:** Chiung-An Chen, Shih-Lun Chen, Hong-Yi Huang, Ching-Hsing Luo

**Affiliations:** 1 Instrumentation Chip Group, Department of Electric Engineering, National Cheng Kung University, Tainan 701, Taiwan; E-Mails: n2895159@mail.ncku.edu.tw (C.-A.C.); chenshihlun@gmail.com (S.-L.C.); 2 Graduate Institute of Electrical Engineering, National Taipei University, Taipei 10478, Taiwan; E-Mail: hyhuang@mail.ntpu.edu.tw (H.-Y.H.)

**Keywords:** asynchronous, micro control unit, multi-sensor, wireless body sensor network

## Abstract

In this work, an asynchronous multi-sensor micro control unit (MCU) core is proposed for wireless body sensor networks (WBSNs). It consists of asynchronous interfaces, a power management unit, a multi-sensor controller, a data encoder (DE), and an error correct coder (ECC). To improve the system performance and expansion abilities, the asynchronous interface is created for handshaking different clock domains between ADC and RF with MCU. To increase the use time of the WBSN system, a power management technique is developed for reducing power consumption. In addition, the multi-sensor controller is designed for detecting various biomedical signals. To prevent loss error from wireless transmission, use of an error correct coding technique is important in biomedical applications. The data encoder is added for lossless compression of various biomedical signals with a compression ratio of almost three. This design is successfully tested on a FPGA board. The VLSI architecture of this work contains 2.68-K gate counts and consumes power 496-μW at 133-MHz processing rate by using TSMC 0.13-μm CMOS process. Compared with the previous techniques, this work offers higher performance, more functions, and lower hardware cost than other micro controller designs.

## 1. Introduction

Recently, the necessity of health monitoring systems using wireless transmission has become prevalent [1–4]. Wireless transmission of health monitoring system data provides comfort and ease in identifying the health conditions of each individual. One of the most practical methods of wireless transmission in health monitoring systems lies on the area of biomedical application in which a wireless body sensor network (WBSN) system is applied. The WBSN system has proven to be an effective method of monitoring health information. In WBSN, the information can be grouped from one wireless body sensor node to another [5,6].

Nowadays, portable wireless body sensor nodes are being developed using a single sensor that can detect only one kind of signal. However, biomedical signals often take different forms such as body temperature [7], heart rate, blood pressure [8], ECG [9], pH value [10], *etc.*, and monitoring such various signals simultaneously would require more than a single body sensor node. The concept of multi-sensor design, which combines different wireless body sensors for flexibility, multi-application, efficiency, and low cost, has been implemented in the literature [11–13]. However, the implementation of multi-sensor architecture imposes a number of design considerations. First, since each sensor handles different kinds of body signals, in order to prevent the occurrence of handshaking problems, the speed of the sensing sensor must synchronize to the clock of the controlling unit. Second, due to the increasing quantity of data from the sensing sensor, which causes bandwidth limitations in the RF module during transmission, the processed data needs some data compression technique to accommodate the whole information. Third, due to the sensitivity of biomedical signals in which the integrity of the information is key, an error correction technique must be implemented. Lastly, since portable wireless body sensor nodes require power to deliver accurate measured result, in order to accommodate a number of body sensor by consuming less power during normal operation, an appropriate power-management supply circuit is needed for power efficiency [14].

Existing general purpose MCUs [15] and DSPs [16] such as the 8051 based controller and C55x are available for possible implementation of the functions discussed above, respectively. In MCU integration design, Amirtharajah [17] presented a micro-power programmable DSP which uses variable precision arithmetic and low-voltage circuits to implement a biomedical detection. Wang [18] designed an 8-bit MCU for a small vocabulary, speaker independent, discrete word speech recognition system. Lee [19] described a smart thermal sensing chip with a MCU design which provides auto-calibration, data compression, and power control for biomedical application. Chen [20] presented a MCU circuit with adaptive low power design for wireless body sensor network. Arm [21] presented a low power design by customizable architecture and reconfigurable instruction set for programmable DSP/MCU core named icyflex1. The mentioned technical works provide great contributions in MCU design, and offer efficient solution for single-sensor of wireless healthcare monitoring systems.

Accordingly, a novel micro control unit is created for wireless multi-sensor body sensor nodes. A novel asynchronous interface is developed to improve the compatibilities of the system which will be composed of devices with different clock domains, which will expand the use range of the wireless body sensor nodes. To increase the detection abilities of each sensor node, we added a multi-sensor control function design to use a single sensor node to detect various biomedical signals by hardware sharing technique. The most important concept in WBSNs is power management. For this reason, a power management technique is developed for reducing power consumption, and furthermore the lossless data encoder is designed to reduce transmission power. Since the reliability is significant in biomedical applications, we developed an error correction coder to prevent loss error from wireless transmissions.

In order to accommodate the above-mentioned necessary functionalities in implementing multi-sensor architecture in biomedical applications, a VLSI implementation of asynchronous multi-sensor micro control unit for WBSNs, which performs more function and control abilities, is proposed in this paper. The proposed architecture is dedicated solely for the multi-sensor application and thus it is low-cost. The control unit of the proposed architecture serves as the core circuit for handling all the processing along with different interfaces. To resolve the issue of handshaking, due to the different clock domains of each sensor, the asynchronous architecture is proposed to ensure proper synchronization. Moreover, the application of the data encoder is included to handle the reduction of transmitted data, which leads to efficient transmission. Lastly, power management and error correction coder (ECC) [22,23] techniques are integrated into the system to minimize power loss and ensure data integrity, respectively.

## 2. System Architecture

**Figure 1.**
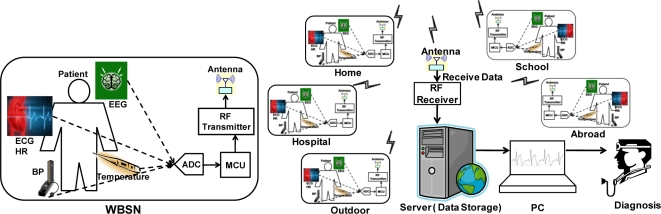
WBSN system scheme for hospital using or health watching application.

Driven by the growing aging population, the prevalence of chronic diseases, and continuously rising healthcare costs, the wireless body sensor network system is undergoing a fundamental transformation, from the conventional hospital-server system to an individual-medical system. Health monitoring systems can be used to collect health data at home, in the hospital, in outdoor scenarios and even abroad. Figure 1 shows the description of a typical wireless body sensor network system that can be used in applications involving biomedical information acquisition, signal processing, data compressing, and wireless transmission. In this figure an individual (*i.e.*, patient) carries various types of sensors such as heart rate (HR), blood pressure (BP), electrocardiogram (ECG), electroencephalogram (EEG), and body temperature. To be able to accurately collect the multiple health information of the individual in a rapid fashion with utmost data integrity, a special kind of micro control unit [24] is needed.

A block diagram of a wireless multi-sensor node for WBSN system is shown in Figure 1. It consists of sensor modules, a multiplexer for data sensor selection, an analog to digital converter (ADC) module, a micro control unit (MCU), a radio frequency (RF) transceiver, and an antenna. The data being sensed from the sensors are transported into ADC, through a multiplexer and fed into the MCU for processing. In this architecture, the data selection is detected simultaneously and the way it is performed is manipulated by the multiplexer, which is controlled mostly by the MCU. Finally, the processed data is transmitted by the antenna through an RF module and stored in a personal computer server for 24-hour display diagnostic.

Furthermore, the MCU is designed to handle two different signal paths; control path and data path. As the MCU is a commander of a WBSN node for control path dominating, with the sensors, the multiplexer, the ADC, and the RF transmitter with antenna are all controlled in orderly fashion in finite state machines (FSM) for power saving in power management (PWM). The various sensors are governed by the control path of the MCU. The sensed data are condensed to process by the data path of MCU.

## 3. MCU Control Path

A wireless body sensor node is formed with the sub-circuits of sensors, ADC, RF and MCU. To group each sub-circuit into a single sensor node, a novel controller is created to improve the system performance and compatibility by asynchronous control, reduce power consumption by power management control and save hardware costs by multi-sensor control. In addition, a finite state machine is designed to control each state to ensure a high performance outcome. The MCU includes a central controller for controlling each module of the wireless body sensor node. Moreover, all functions including multi-sensor, ADC, DE, ECC, and RF of the wireless sensor node are controlled by the central controller. Each module of the system is driven by a power source and function control signals from the MCU. A finite state machine (FSM) is designed to detect control states of the ongoing activity of each sensor node. This design enables the system to automatically compute when the user tries to input the sensor signals through the interface.

**Figure 2.**
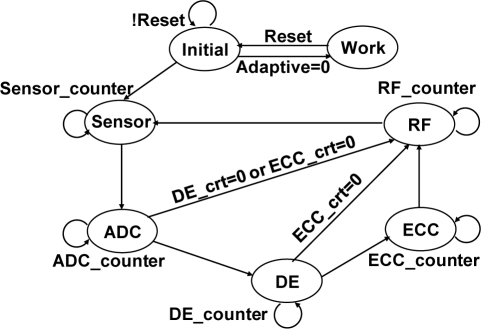
Main FSM of the MCU.

**Figure 3.**
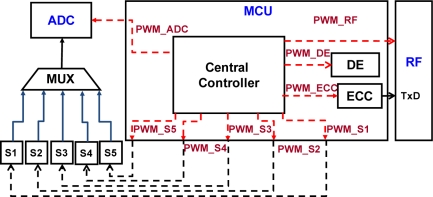
PWM control path for all devices.

As shown in Figure 2, the main FSM of the MCU unit consists of seven states. When the adaptive status lies in the zero value, the working state is activated, which means all functions or state conditions of the whole system are powered on. On the contrary, when the status goes high, the normal flow of state signals is activated, by which each state such as sensor, ADC, DE, ECC, and RF functions stay in off condition until the previous state has finished its function. Following Figure 2, a detail illustration with each state represented by its actual device is discussed in Figure 3.

### 3.1. Power Management

The power consumption of the whole WBSN system is reduced by power management [21]. As shown in Figure 3, the proposed micro control unit handles all the major operations in interfacing the necessary devices such as ADC, DE, ECC, and sensors into the central controller, thus regardless of what operation is performed the power status of the node is always turned on. In order to optimize the power handling within the MCU, an effective power management scheme is necessary for these devices. In this work, the main purpose of power management technique is to control the power state of a system to reduce power consumption depending on system state condition. As can be seen from Figure 3, unless the adaptive status is enabled to enter into working state, each part performs only when it is powered on leaving the rest (unused) state off until going to the next-state. For example, when the power management controller stays on DE-state, as shown in Figure 2. There is only the DE function working and powered on. Other functions are sleeping and powered off. Each function is powered on only when it is necessary to be used. With this, the behavior of the system, in power consumption perspective, is handled by the power management design. Furthermore, the task of central controller is not limited only to control sensor selection and data compression, but also control all the power of the MCU. A low power controller, which is a sub-FSM circuitry inside the central controller, is included for handling low power activity.

### 3.2. Multi Sensor Controller

In order to provide more options in the multi-sensor for bio-medical detection, the sensor controller should perform as the command-processor for those sensors. The MCU sends instructions to the multiplexer and makes each sensor perform according to the hierarchy of the operation. If more than two sensors are working simultaneously, the data from different sensors is passed-through correctly. Each sensor has its own buffer for storing the sensed data through selection procedure of the multiplexer. The sensor-select option enables the operation of sensor-multiplexer pair, which ensures that the output data is processed independently. The sensor-select option is controlled by external operation. Assuming that the external operation selects two sensors, the MCU will process the transmitted data and stored in the buffer. After which, the MCU then continues to process with the next sensor in accordance to the hierarchy of operation.

**Figure 4.**
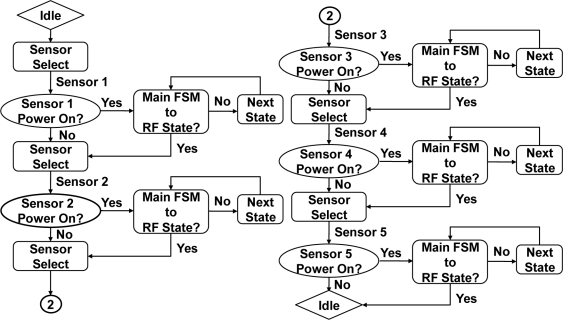
Control flow of multi sensor controller.

Regardless of the number of sensors working simultaneously the controller can be able to handle each sensor. Referring to Figure 4, the idle-state represents a normal condition. Then sensor-select-state received order from MCU detecting with sensor 1. If power on is confirmed by MCU then accessing to sensed data processing. If power off then goes back to Sensor-Select state with sensor 2 checked. Repeating steps with each sensor intern, till sensor 5 checked and back to idle-state. When sensor selection is performed, the flow to the next sensor in the state is determined by sensor-select. Further, sensor-next-state condition relies on the command from the MCU, and is with accordance with the sensor-select signal.

## 4. MCU Data Path

**Figure 5.**
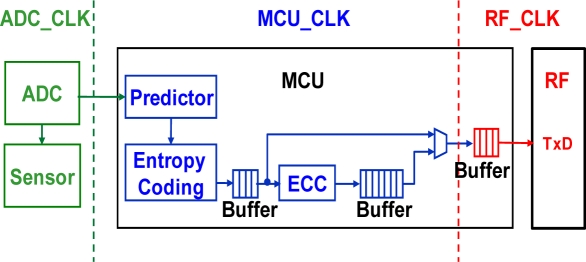
Three clock domains of the wireless body sensor node.

To decrease the transmission data, the data encoder for lossless compression of various biomedical signals is designed. In addition, an error correct coder circuit is also created for the proposed multi-sensor wireless body sensor network application. By the creative combination of the data encoder and error correct coder, the MCU design gains both benefits of lower data rate and higher communication reliability. Figure 5 shows the data flow of the wireless body sensor node. Since biomedical signals are quite slow (*i.e.*, 1 to 100 Hz [25]), the variations of the adjacent values of body signals are very small. For a 12-bit ADC used for temperature signal from the human body, the resulting values are way too large to be stored or to be transmitted. After the signal passes through the predictor module, the difference values extracted from the original temperature data are measured close to zero. Base from this characteristic, the concept of Huffman coding [26] was chosen to handle the data compression algorithm. The probability of Huffman encoding will be decided by the characteristics of different biomedical signals. Furthermore, the ECC is included for particular characteristics to make sure that 100% of data is correctly transmitted in RF transmission. After transmission through the RF transmitter and reception by the RF receiver, the encoded data stream will be decoded by ECC decoding, entropy decoding, and prediction refining.

### 4.1. Asynchronous Interface

To ensure that the signals coming from the ADC, and transmitted through RF module matches with full integrity with the original signal, the proposed MCU employs an asynchronous interface method which connects the MCU to its respective input and output module. Figure 5 shows the typical clock domain illustration of the wireless sensor node. In this figure, each of the three major blocks, ADC, MCU, and RF have separate clock domain operation. The data accessed to go through these three blocks would require an asynchronous technique function to prevent the occurrence of handshake problems. By using a buffer to assist the data transmission, the signals coming from the ADC are carried into the ADC_CLK block. The MCU takes samples from these signals and stores them into a register that resides in the MCU_CLK. Finally the MCU has to transfer these signals into the buffer, which also resides in the MCU_CLK.

**Figure 6.**
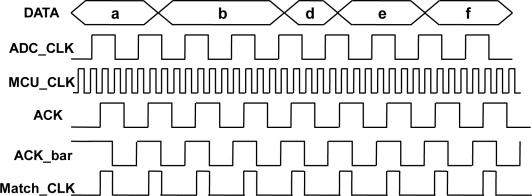
Example of handshake between asynchronous interfaces.

Figure 6 shows the clocking signal example of handshaking activity between each asynchronous interface. First, the highest frequency of the clock domain is selected as a sampling clock signal which is much faster than the others. This sampling clock is used to perform the sampling of other clock domain signals which is represented as an ACK signal. Next, the ACK signal is delayed one sampling clock as ACK_bar signal. After the sampling and delay steps, both the ACK and ACK_bar signals are in sampling clock domain which is represented as MCU_CLK. Finally, the ACK and ACK_bar signals are computed by a NAND function which eventually produces the Match_CLK result. The Match_CLK signal indicates the MCU when to input the signals from different clock domains.

### 4.2. Data Encoder

The data encoder consists of two function blocks: predictor and entropy encoder. It basically performs the compression of the transmitted data which brings much improvement on power consumptions through shorting transceiver power on time.

**Figure 7.**
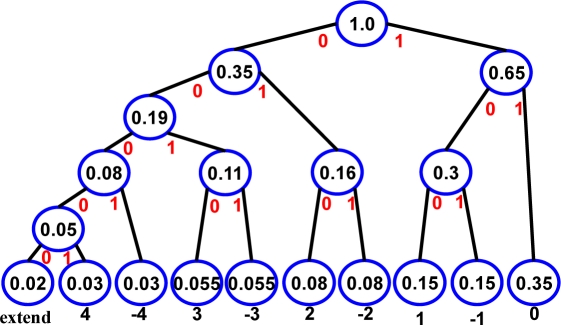
The Huffman coding tree.

*(1) Predictor*: The predictor block outputs the different value from two continuous biomedical signals, and sends these values into the entropy encoder. The predictor is designed for detecting the different values between continuous biomedical signals. One common example is the variation of body temperature which is very small in quantity. In this way, the probabilities are concentrated on small difference values. On the other hand, if the biomedical signals contain more variations like ECG, the probabilities are dispersed to each difference value. The predictor consists of two registers and one sub-tractor. In addition, the input data are connected to 12-bit ADC output. This output signals are represented as 1-bit sign and single 12-bit difference signal. The sign and difference signals are sent to entropy encoder for compression.
*(2) Lossless Compression*: Entropy Encoder: The entropy encoder is designed using entropy coding, which is a technique for lossless data compression to achieve compact data representation by taking advantage of the statistical characteristics of data. Today, the most practiced entropy coding technique is Huffman coding [20]. It is widely used in various data compression applications and has been already adopted in many international standards. Huffman coding was selected because it provides minimum encoding cost when the original signal has the predicable distribution. The Huffman coding translates input data, often in a fixed-length format, into codes of variable lengths. The basic concept behind Huffman coding is to represent more frequent data by shorter codes and less frequent data by longer codes. Therefore, the average code length is expected to be shorter than that of the fixed-length representation. Figure 7 shows the tree architecture of the Huffman coding. It is shown that the variable length code (VLC) is fixed by a probability of predictor values. Since the range of signals in biomedical application is not widely changed per second, the highest probability value approximates nearly to 35% and is fixed at the shortest length when difference value is zero. Moreover, the longest length with the biggest difference value is assigned by extend code.

Table 1 presents the Huffman coding tree distribution format. Assuming an ADC of 12-bit (ADS7842) [27] is used, without Huffman coding, the output fixed-length has a value equal to the resolution of the ADC, which in this case is 12. For the most part, data encoder with Huffman coding decreases the transport bits effectively. The date encoder is designed for data compression. In order to validate the proposed classification algorithm and compared, the MIT-BIH Arrhythmia [28] data base have been used. The ECG signals were digitized through sampling at 360 samples per second, quantized and encoded in 11 bits. In this paper, we used MIT-BIH pattern as an example to value the compression rate of data encoder. As shown in Table 2, the compression rate (CR) value is over than 2.26, which means less than half of ECG transmission data are reduced by data encoder.

### 4.3. Error Control Coding (ECC) Circuit

Error control coding is widely used to increase the reliability of transmission systems. It is designed for decreasing the transmission error rate by adding some remainder codes into the original stream. First, a generator polynomial function g(X) is assumed. The product function p(X) is the message polynomial function u(X) multiplied by X^n-k^ as:

(1) $$\text{p}(\text{X})={\text{X}}^{\text{n}-\text{k}}\text{u}(\text{X})$$

where n-k is the degree of g(x). After which, the product function p(X) is by g(X) to obtain the remainder b(X). Thus, the ECC code can be generated by p(X) merging b(X) expression as:

(2) ECC(X)=b(X)+p(X)

The ECC circuit can be accomplished with a division circuit which is a linear (n-k)-stage shift register with feedback connections based on the generator polynomial is given by:

(3) $$\text{g}(\text{X})=1+{\text{g}}_{1}\text{X}+{\text{g}}_{2}{\text{X}}^{2}+\ldots +{\text{g}}_{\text{n}-\text{k}-1}{\text{X}}^{\text{n}-\text{k}-1}+{\text{X}}^{\text{n}-\text{k}}$$

**Figure 8.**
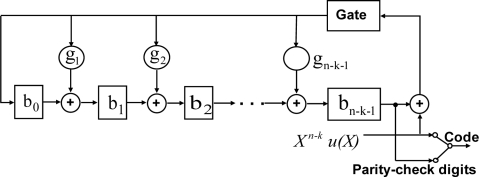
Architecture of ECC encoder.

Figure 8 shows the architecture of ECC encoder circuit composed of registers and logical circuits. With the gate turn on, the k information digits u_0_, u_1_, …, u_k-1_ are shift into the circuit and simultaneously into the communication channel. In polynomial form is shown as:

(4) $$\text{u}(\text{X})={\text{u}}_{0}+{\text{u}}_{1}\text{X}+{\text{u}}_{2}{\text{X}}^{2}+\ldots +{\text{u}}_{\text{k}-1}{\text{X}}^{\text{k}-1}$$

The u(X) is shifted into the circuit from the front end is equivalent to polynomial function u(X) by X^n-k^. Until the complete message enters the circuit, the remainder is composed by the n-k digits in the register and thus they are the parity-check digits. The next-step breaks the feedback connection by turning off the gate. Finally, the parity-check digits are shifted out and send into the channel. These n-k digits b_0_, b_1_, …, b_n-k-1_, together with the k information digits, form a complete ECC code. The ECC decoder can automatically check the receiving stream. If the steam is correct, it will be sent out directly. On the other hand, the ECC decoder will correct error bits immediately and then send out the correct stream and error information. This error control coding design improves the reliability of biomedical signals for WBSN systems.

### 4.4. Data Stream

**Figure 9.**
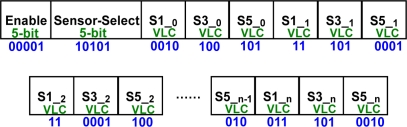
The data stream of the sensor controller.

The MCU has to prevent the data stream from losing the data of each sensor. As sensor-select sets down, each sensor will get its own data stream which is a variable length code (VLC). An example is depicted in Figure 9. Since sensor one, three, and five are set on working state, the Sensor-Select prints 10101. The first data comes from S1 is 0010, and stored in the buffer. The same goes to the rest of the enabled sensor. Because of the DE function, the data prints out with different length code. Regardless how many data in VLC comes in, the data stream control handles transportation of every single data item correctly.

## 5. Experimental Section

**Figure 10.**
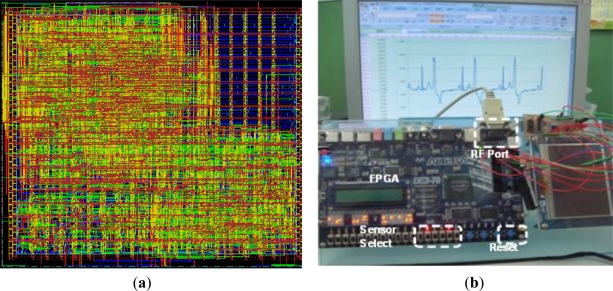
(**a**) MCU layout in TSMC013 process. (**b**) MCU achived by FPGA emulation.

In order to realize the WBSN node in hardware applications, we emulated the microcontrol unit design on a FPGA board. The MCU design was implemented by verilog code, simulated by ncverilog tool, and synthesized by design compiler tool. Before the layout of the MCU has been realized by SoC-encounter, the design was initially emulated in Altera FPGA EP2C70F896C6 core. This design was implemented by TSMC 0.13-μm CMOS generic logic process technology. The core area is measured in 0.014 mm^2^ with a power consumption of 496 μW operating at 133 MHz frequency. Figure 10(a) displays the layout photograph of the MCU.

Table 3 lists the specifications of this work and other proposed MCU designs. Including a micro-power DSP design [17] synthesized by 0.6-μm process or MCU design for biomedical application [20] by 0.18-μm process are both different from this work. First of all, a multiple sensors function is performed in this work. For comparison purposes, we convert its core area to 0.13-μm, which allows us to gain a normalized area corresponding to a 0.13-μm process. As a result of normalization, the normalized areas of [17,20] or [21] are 70.9, 4.96, or 31.85 times of this work, resepectively. This work achieves 133-MHz operating frequency, higher than 1.2-KHz, 8.2-MHz, 75-MHz, and 100-MHz of other works. The gate count of this work is 2.68-K, much less by 80% than other precise MCU cores. In order to show power consumption with different operation frequency, the power consumptions of this work operating in 1-MHz, 75-MHz, 100-MHz, and 133-MHz are 3.7 μW, 280 μW, 375 μW, and 496 μW.

Figure 10(b) shows the FPGA emulation of MCU. This system consists of an RF (RS232 protocol) and a FPGA development board. The MCU is compiled by Quartus II and downloaded into ATERA DE2-70 FGPA. The FPGA receives the imported MIT-BIH ADC 11-bit data and process it by the compress and ECC functions of the MCU which then downloaded it into the FPGA. The compressed data from the FPGA output is transmitted from the RF port by the RS232 protocol. After the computer receives the transmitted data, the decoded program, which is written in C language, is decrypted by the decoding algorithms. The result of the decryption is guaranteed to be a 100% correct and refined MIT-BIH ECG database [28].

Table 4 shows the comparison result of different functions of a wireless body sensor node using the MIT-BIH ECG database. For nomal node design including ADC (ADS7842) and RF tranceiver (Zigbee CC2430) [27], the power consumed is 4.5 mW and 81 mW, respectively. Further, the produced wireless transmission error rate is given as 1% bps. In order to obtain the contribution of each creative part, the proposed MCU design was implemeted with creative functions. The WBSN system with power management, DE, and ECC creative function modes and without any mode design are listed in Table 4. The benefit of including the data encoder (DE) not only reduces its data rate but also describes the trasmission data, which means that part of transmitting power is saved. In this case, the creative funtion mode includes power management and data encoder (PWM + DE) that reduced power comsuption by 97.8% and decreased the data rate by 55% more than without the MCU design. Moreover, the ECC design is created to prevent RF transmission errors. In this case, although the ECC creative function mode increases the data rate to 4,620 bit/s, the data rate is still less than that without MCU design with 4,640 bit/s due to the benefit of the data encoder. Futhermore, the creative ECC design can efficeintly reduce 1% bps error rate in this case, which improves the reliability for wireless biomedical applications significantly. To further improve the performance, in this work mode combined with creative DE, and ECC functions, it guarantees that the gains of each creative function are fully utilized.

## 5. Conclusions

In this paper, we present an asynchronous micro ontrol unit for wireless body multi-sensor networks. This design provides multiple functions and advanced system quality. The operating frequency of the MCU is 133 MHz, with a power consumption of only 496-μW. As compared with wireless sensor nodes without the presence of MCU, this work has the benefits of lower power consumption, less error rate, and faster data rate. These characteristics are the basic requirements for developing a wireless body sensor network (WBSN) system.

## Figures and Tables

**Table 1.** The Entropy Coding Table.

**Difference Value**	**Probability**	**VLC**	**Length**

0	0.35	11	2
1	0.15	100	3
−1	0.15	101	3
2	0.08	010	3
−2	0.08	011	3
3	0.055	0010	4
−3	0.055	0011	4
4	0.03	00001	5
−4	0.03	0001	4
Extend	0.02	00000	5

**Table 2.** The Data Encoder Compression Rate.

**MIT-BIH Pattern No.**	**Original (KB)**	**Compression Ch1 (KB)**	**Compression Ch2 (KB)**	**Compression Rate (CR)**

101	1,696	383	309	2.45086
112	1,696	384	337	2.35228
115	1,696	357	327	2.47953
121	1,696	351	398	2.26435
201	1,696	341	350	2.45441
205	1,696	345	330	2.51259
231	1,696	367	337	2.40909

**Table 3.** MCU Comparison with Proposed Work.

	**Sensors No.**	**Process**	**Operation Freq. (Hz)**	**Gate Count**	**Chip Area**	**Normalized Area**

[17] JSSC ‘04	1	0.60-μm	1.2 K	190 K	3.200 mm^2^	70.90
[18] ICASSSP ‘04	1	0.50-μm	8.2 M	Non	Non	N/A
[19] IEICE ‘08	1	0.18-μm	100 M	Non	<1 mm^2^	N/A
[20] ISJ ‘09	1	0.18-μm	100 M	13.4 K	0.134 mm^2^	4.96
[21] JSSC ‘09	1	0.13-μm	75 M	110 K	0.430 mm^2^	31.85
This work	4	0.13-μm	133 M	2.68 K	0.014 mm^2^	1

Note: The area is normalized to 0.13-μm process by Area_process_ × (0.13/Process)^2^ or gate counts where Area_process_ denotes the silicon area in their original process.

**Table 4.** Functions Comparison of WBSN.

**Mode**	**Functions**	**Data Rate (bit/s)**	**Transmission Error Rate**	**Refined Correct Rate**

Without MCU	None	2,640	1%	99%
PWM	PWM	2,640	1%	99%
PWM+DE	PWM, DE	1,168	1%	99%
PWM+ECC	PWM, ECC	4,620	1%	100%
This Work (PWM + DE + ECC)	PWM, DE, ECC	2,044	1%	100%
Mode	Functions	Data Rate (bit/s)	Transmission Error Rate	Refined Correct Rate
